# Current Sensor Fault Diagnosis Based on a Sliding Mode Observer for PMSM Driven Systems

**DOI:** 10.3390/s150511027

**Published:** 2015-05-11

**Authors:** Gang Huang, Yi-Ping Luo, Chang-Fan Zhang, Yi-Shan Huang, Kai-Hui Zhao

**Affiliations:** 1 School of Traffic and Transportation Engineering, Central South University, Changsha 410075, China; E-Mails: ypluo@csu.edu.cn (Y.-P.L.); zhaokaihuicn@gmail.com (K.-H.Z.); 2 College of Electrical and Information Engineering, Hunan University of Technology, Zhuzhou 412007, China; E-Mail: zhangchangfan@263.net; 3 Hunan CSR Times Electric Vehicle Co., Ltd, Zhuzhou 412007, China; E-Mail: huangys@teg.cn

**Keywords:** permanent magnet synchronous motor (PMSM), extended flux linkage, sliding mode observer (SMO), current sensor, fault diagnosis

## Abstract

This paper proposes a current sensor fault detection method based on a sliding mode observer for the torque closed-loop control system of interior permanent magnet synchronous motors. First, a sliding mode observer based on the extended flux linkage is built to simplify the motor model, which effectively eliminates the phenomenon of salient poles and the dependence on the direct axis inductance parameter, and can also be used for real-time calculation of feedback torque. Then a sliding mode current observer is constructed in αβ coordinates to generate the fault residuals of the phase current sensors. The method can accurately identify abrupt gain faults and slow-variation offset faults in real time in faulty sensors, and the generated residuals of the designed fault detection system are not affected by the unknown input, the structure of the observer, and the theoretical derivation and the stability proof process are concise and simple. The RT-LAB real-time simulation is used to build a simulation model of the hardware in the loop. The simulation and experimental results demonstrate the feasibility and effectiveness of the proposed method.

## 1. Introduction

Permanent magnet synchronous motors (PMSMs) can efficiently satisfy the requirements of railway vehicles, including power quality, energy consumption and control characteristics thanks to their high efficiency, high ratio of torque to weight, high power density and faster response [1,2,3]. Germany, France, Japan and other countries have successively developed PMSM traction driven systems. Recently the first high-speed train equipped with a permanent magnet traction system came off the assembly line in CSR Qingdao Sifang Co., Ltd. Permanent magnet synchronous traction systems have taken the lead as the future of rail traction drive development [4,5]. However, due to China’s vast territory and large trains’ operation span, permanent magnet synchronous traction systems are susceptible to the influence of the external environment that leads to fault risks, which could directly result in system torque performance deterioration and seriously affect the safe operation of trains. Therefore, it’s very important to carry out research of online condition monitoring for permanent magnet synchronous traction motors to reduce the risk of faults.

A typical PMSM driven system requires at least two alternating-current sensors, and failure of either one or the two will lead to performance degradation [6], so increasing concerns about sensor faults have led some researchers to focus their efforts on developing sensor fault diagnosis methods. However, most of the previous studies focus on inverter faults or motor body faults [7,8,9,10,11,12,13,14,15], and hardly any research on sensor fault diagnosis of PMSM can be found. Current, position and dc-link voltage sensor fault diagnosis for a PMSM driven system are studied in [16], but its current sensor fault diagnosis method is offline. A nonlinear parity relation method for diagnosis of additive faults for virtual sensors for d-q axis currents is studied in [17]; it can identify the faults appearing in the phase current sensors through abnormal changes in the d-q axis currents, but this method cannot provide specific information about which phase current sensor is faulty. A fault diagnosis structure of position and phase current sensors faults of a PMSM driven system is studied in [18]; the method is based on two interconnected observers: an Extended Kalman Filter and a Model Reference Adaptive System observer. Current, position and dc-link voltage sensor fault diagnosis for a PMSM driven system based on an Extended Kalman Filter is studied in [6], but the performance based on the Extended Kalman Filter algorithm will deteriorate at low speed, will be affected to a certain extent by parameter changes and is not sensitive to slow-variation faults. An adaptive observer method for detection and isolation of abrupt gain faults and offset faults for phase current sensors is studied in [19,20], but they did not consider the slow-variation faults which are difficult to observe, and only give the fault residuals of αβ axis virtual current sensors, then detect the phase sensor faults through logical judgments, but this easily causes misjudgments because the threshold is set larger, and the motor model adopted by the tmethod has serious salient features which are inconvenient for the observation of PMSM status.

Therefore, on the basis of literature [19,20], a current sensor fault detection method is proposed for a torque closed-loop control system of an interior PMSM based on a sliding mode control method due to its good robustness to parameter perturbation, external disturbances and inaccurate mathematical models [21,22]. First a sliding mode observer based on the extended flux linkage is designed to simplify the motor model, which effectively eliminates the phenomenon of salient poles and the dependence of the direct axis inductance parameter, and can also be used for online calculation of feedback torque. Then a sliding mode current observer is constructed in αβ coordinates to generate fault residuals of the phase current sensors. The method can accurately identify abrupt gain faults and slow-variation offset faults in real-time. The RT-LAB real-time simulation is used to build a simulation model of the hardware in the loop. The simulation and experimental results demonstrate the feasibility and effectiveness of this method.

## 2. Mathematical Mode of the IPMSM

The voltage and flux equations for IPMSM in the rotating dq-reference frame are as follows [23]:

(1) $$\left[\begin{array}{l} u_{d}\\ u_{q} \end{array}\right]=\left[\begin{array}{l} R_{s}+DL_{d}\;-\omega L_{q}\;\\ \;\omega L_{d}\;R_{s}+DL_{q}\; \end{array}\right]\left[\begin{array}{l} i_{d}\\ i_{q} \end{array}\right]+\omega \left[\begin{array}{l} 0\\ \psi_{r} \end{array}\right]$$

(2) $$\left\{ \begin{array}{l} \psi_{d}=L_{d}i_{d}+\psi_{r}\\ \psi_{q}=L_{q}i_{q} \end{array}\right.$$

In Equations (1) and (2), *R_s_* is the stator resistance, *L_d_* and *L_q_* are the *d* and *q* axis stator inductances, *u_d_* and *u_q_* are the *d* and *q* axis stator voltages, *i_d_* and *i_q_* are the *d* and *q* axis stator currents, *ψ_r_* is the permanent magnet flux linkage, *ψ_d_* and *ψ_q_* are the *d* and *q* axis stator fluxes: ω is the electrical rotor speed, and *D* is the differential operator.

By using coordinate inversion, the voltage Equation (1) in the stationary reference αβ frame are transformed to be:

(3) $$\left[\begin{array}{l} u_{\alpha }\\ u_{\beta } \end{array}\right]=R_{s}\left[\begin{array}{l} i_{\alpha }\\ i_{\beta } \end{array}\right]+D\left[\begin{array}{l} L_{1}+L_{2}\text{cos2}\theta \;L_{2}\text{sin2}\theta \;\\ \;L_{2}\text{sin2}\theta \;L_{1}-L_{2}\text{cos2}\theta \; \end{array}\right]\left[\begin{array}{l} i_{\alpha }\\ i_{\beta } \end{array}\right]+\omega \psi_{r}\left[\begin{array}{l} -\text{sin}\theta \\ \text{cos}\theta \end{array}\right]$$

where *L*_1_ = (*L_d_ + L_q_*)/2; *L*_2_ = (*L_d_ −* *L_q_*)/2; *u_α_* and *u_β_* are the α and β axis stator voltages, *i_α_* and *i_β_* are the α and β axis stator currents, θ is the electrical rotor angular position. Items with 2θ in Equation (3) show the salient features of IPMSM, and it is inconvenient for the observation of the PMSM status.

In order to eliminate the salient pole phenomenon, the voltage Equation (1) can be rewritten as:

(4) $$\left[\begin{array}{l} u_{d}\\ u_{q} \end{array}\right]=\left[\begin{array}{l} R_{s}+DL_{q}\;-\omega L_{q}\;\\ \;\omega L_{q}\;R_{s}+DL_{q}\; \end{array}\right]\left[\begin{array}{l} i_{d}\\ i_{q} \end{array}\right]+\omega \left[\begin{array}{l} \;0\\ \psi_{r}+(L_{d}-L_{q})i_{d} \end{array}\right]+\left[\begin{array}{l} (L_{d}-L_{q})Di_{d}\\ \;0 \end{array}\right]$$

where: $\left[\begin{array}{c} 0\\ \psi_{r}+(L_{d}-L_{q})i_{d} \end{array}\right]$ is the extended flux linkage in the rotating *dq*-reference frame, the inductance matrix of Equation (4) is a symmetric matrix, and only contains *R_s_* and *L_q_*. By using coordinate inversion, the voltage Equation (4) in the stationary reference αβ frame are transformed to be:

(5) $$\left[\begin{array}{l} u_{\alpha }\\ u_{\beta } \end{array}\right]=R_{s}\left[\begin{array}{l} i_{\alpha }\\ i_{\beta } \end{array}\right]+DL_{q}\left[\begin{array}{l} i_{\alpha }\\ i_{\beta } \end{array}\right]+\omega \left[\begin{array}{l} \psi_{r}+(L_{d}-L_{q}\text{)}i_{d}\;0\;\\ \;0\;\psi_{r}+(L_{d}-L_{q}\text{)}i_{d}\; \end{array}\right]\left[\begin{array}{l} -\text{sin}\theta \\ \text{cos}\theta \end{array}\right]$$

where ***u**_αβ_* is the voltage vector defined as ***u****_αβ_* = [*u_α_ u_β_*]^T^, *i_αβ_* is the current vector defined as ***u****_αβ_* = [*u_α_ u_β_*]^T^, *i_αβ_* is the current vector defined as *i_αβ_*= [*i_α_ i_β_*]^T^, *ψ_ext,αβ_* is the extended flux linkage vector in the stationary reference αβ frame and is given by:

(6) $$\psi_{ext,\alpha \beta }=\left[\begin{array}{l} \psi_{ext,\alpha }\\ \psi_{ext,\beta } \end{array}\right]=\left[\begin{array}{l} \psi_{r}+(L_{d}-L_{q}\text{)}i_{d}\;0\;\\ \;0\;\psi_{r}+(L_{d}-L_{q}\text{)}i_{d}\; \end{array}\right]\left[\begin{array}{l} \cos\theta \\ \sin\theta \end{array}\right]$$

Equation (5) can be rewritten as:

(7) $$u_{\alpha \beta }=R_{s}i_{\alpha \beta }+DL_{q}i_{\alpha \beta }+D\psi_{ext,\alpha \beta }$$

According to the Equation (6), Equation (8) can be obtained [24]:

(8) $$D\psi_{ext,\alpha \beta }=\omega J\psi_{ext,\alpha \beta }$$

The electromagnetic torque equation can be described as:

(9) $$T_{e}=\frac{3}{2}n_{p}\psi_{s}^{T}J^{T}i_{\alpha \beta }$$

where *J* is the vector defined as $J=\left[\begin{array}{l} 0\;-1\\ 1\;0 \end{array}\right]$, *ψ_s_* is the stator flux linkage vector, *n_p_* is the number of pole pairs.

## 3. SMO Design Based on Extended Flux Linkage

According to Equations (7) and (8), a state space representation can be described as follows [24]:

(10) $$\left\{ \begin{array}{l} D\psi_{ext,\alpha \beta }=\omega J\psi_{ext,\alpha \beta }\\ y_{1}=\omega J\psi_{ext,\alpha \beta }=u_{\alpha \beta }-R_{s}i_{\alpha \beta }-DL_{q}i_{\alpha \beta } \end{array}\right.$$

where *ψ_ext,αβ_* is the state variable, *y*_1_ is the output vector. The sliding mode observer based on extended flux linkage is designed as:

(11) $$\left\{ \begin{array}{l} D{\hat{\psi }}_{ext,\alpha \beta }=\omega J{\hat{\psi }}_{ext,\alpha \beta }+K\left|e_{ext,\alpha \beta }\right|\mathrm{sgn}(e_{ext,\alpha \beta })\\ {\hat{y}}_{1}=\omega J{\hat{\psi }}_{ext,\alpha \beta } \end{array}\right.$$

where ${\hat{\psi }}_{ext,\alpha \beta }$ and ${\hat{y}}_{1}$ are the observations of *ψ_ext,αβ_* and *y*_1_. The chattering caused by the constant rate reaching law based on constant switching control is larger, in order to make the approach speed and the change of the state vector of the system associated in reaching the sliding mode switching surface movement phase, the absolute value of state error is used as switching control item in the observer to reduce the chattering and improve the dynamic response speed of the system. *K*|*e_ext,αβ_*|sgn(*e_ext,αβ_*) is designed as the sliding mode switching control function of the variable rate reaching law, *K* is the matrix of sliding mode gain defined as $K=\left[\begin{array}{l} k_{\text{1}}\;0\\ \text{0 }k_{\text{2}} \end{array}\right]$, *k*_1_ > 0 and *k*_2_ > 0 are pending values. *e_ext,αβ_* is the error defined as $e_{ext,\alpha \beta }=\left[\begin{array}{l} e_{ext,\alpha }\\ e_{ext,\beta } \end{array}\right]=\left[\begin{array}{l} \psi_{ext,\alpha }-{\hat{\psi }}_{ext,\alpha }\\ \psi_{ext,\beta }-{\hat{\psi }}_{ext,\beta } \end{array}\right]$ spn(·) is the sign function.

*s* is the sliding surface defined as $s=\left[\begin{array}{l} s_{1}\\ s_{2} \end{array}\right]=e_{ext,\alpha \beta }$, and because $\omega J\psi_{ext,\alpha \beta }=\omega \left[\begin{array}{l} -\psi_{ext,\beta }\\ \psi_{ext,\alpha } \end{array}\right]$, then:

(12) $${\dot{s}}_{1}={\dot{e}}_{ext,\alpha }={\dot{\psi }}_{ext,\alpha }-{\dot{\hat{\psi }}}_{ext,\alpha }=-\omega \psi_{ext,\beta }+\omega {\hat{\psi }}_{ext,\beta }-k_{1}\left|e_{ext,\alpha }\right|\mathrm{sgn}(e_{ext,\alpha })=-\omega e_{ext,\beta }-k_{1}\left|e_{ext,\alpha }\right|\mathrm{sgn}(e_{ext,\alpha })$$

(13) $${\dot{s}}_{2}={\dot{e}}_{ext,\beta }={\dot{\psi }}_{ext,\beta }-{\dot{\hat{\psi }}}_{ext,\beta }=-\omega \psi_{ext,\alpha }+\omega {\hat{\psi }}_{ext,\alpha }-k_{2}\left|e_{ext,\beta }\right|\mathrm{sgn}(e_{ext,\beta })=-\omega e_{ext,\alpha }-k_{1}\left|e_{ext,\beta }\right|\mathrm{sgn}(e_{ext,\beta })$$

The design goal of the observer is to make the observation error converge to zero by selecting the appropriate *k*_1_ and *k*_2_.

Proof:

Select a non-negative Lyapunov function which can be expressed as:

(14) $$V=\frac{1}{2}(s_{1}^{2}+s_{2}^{2})$$

According to Equations (12) and (13), then:

(15) $$\dot{V}=s_{1}{\dot{s}}_{1}=s_{2}{\dot{s}}_{2}=-\omega e_{ext,\alpha }e_{ext,\beta }--k_{1}{e_{ext,\alpha }}^{2}+\omega e_{ext,\alpha }e_{ext,\beta }-k_{2}{e_{ext,\beta }}^{2}=-(k_{1}{e_{ext,\alpha }}^{2}+k_{2}{e_{ext,\beta }}^{2})\leq 0$$

Based on the Lyapunov stability theory, the designed sliding mode observer is proved to be asymptotically stable. After the state of the system reaches the sliding mode surface, *e_ext,αβ_* = 0, then:

(16) $$\omega Je_{ext,\alpha \beta }=\omega J(\psi_{ext,\alpha \beta }-{\hat{\psi }}_{ext,\alpha \beta })=0$$

According to Equations (10) and (16), an observation of the stator flux linkage based on the stator voltage model can be obtained as follows:

(17) $${\hat{\psi }}_{s}=\int (u_{\alpha \beta }-R_{s}i_{\alpha \beta })dt=L_{q}i_{\alpha \beta }+{\hat{\psi }}_{ext,\alpha \beta }$$

Then put Equation (17) into Equation (9), an observation of the electromagnetic torque can be gotten as the following:

(18) $${\hat{T}}_{e}=\frac{3}{2}n_{p}{\hat{\psi }}_{s}^{T}J^{T}i_{\alpha \beta }=\frac{3}{2}n_{p}{\left(L_{q}i_{\alpha \beta }+{\hat{\psi }}_{ext,\alpha \beta }\right)}^{T}J^{T}i_{\alpha \beta }$$

Despite the observation of the extended flux linkage may be influenced by *q* axis inductance, the study found that the observed stator flux linkage through our scheme is not influenced by *dq* axis inductance. The stator flux linkage of Equation (17) both retained the robustness of the voltage model method, and overcame the pure integral problems of the voltage model method. Then the electromagnetic torque observation of Equation (18) is also not influenced by *dq* axis inductance and variation of the permanent magnet flux linkage, thus the accuracy of the torque observation can be ensured [24].

## 4. Fault Residual Generation Based on SMO

According to Equation (5), the state space model of PMSM can be rewritten as:

(19) $$D\left[\begin{array}{l} i_{\alpha }\\ i_{\beta } \end{array}\right]=-\frac{R_{s}}{L_{q}}\left[\begin{array}{l} i_{\alpha }\\ i_{\beta } \end{array}\right]+\frac{1}{L_{q}}\left[\begin{array}{l} u_{\alpha }\\ u_{\beta } \end{array}\right]+\frac{\omega }{L_{q}}\left[\begin{array}{l} \psi_{ext,\beta }\\ -\psi_{ext,\alpha } \end{array}\right]$$

where: *x* = [*i_α_ i_β_*]^T^, *u* = [*u_α_ u_β_*]^T^, $A=\left[\begin{array}{l} -\frac{R_{s}}{L_{q}}\text{ 0}\\ \;0\;-\frac{R_{s}}{L_{q}} \end{array}\right]$, $B=\left[\begin{array}{l} \frac{1}{L_{q}}\text{ 0}\\ \;0\;\frac{1}{L_{q}} \end{array}\right]$, $C=\left[\begin{array}{l} 1\;0\\ 0\;1 \end{array}\right]$, $F=\left[\begin{array}{l} \text{ 0 }\frac{\omega }{L_{q}}\\ \;-\frac{\omega }{L_{q}}\;0 \end{array}\right]=-\frac{\omega }{L_{q}}J$.

Therefore the equation of motor model with the system uncertainties in the case of sensor fault can be described as follows:

(20) $$\left\{ \begin{array}{l} \dot{x}(t)=Ax(t)+Bu(t)+F\psi_{ext,\alpha \beta }+Dd(x,u,t)\\ y_{2}(t)=Cx(t)+Gf_{s} \end{array}\right.$$

where: *f_s_* is the sensor fault vector of αβ axis stator current defined as *f_s_* = [*f_sα_ f_sβ_*]^T^, $G=\left[\begin{array}{l} 1\;0\\ 0\;1 \end{array}\right]$. $D=\left[\begin{array}{l} 1\;0\\ 0\;1 \end{array}\right]$, *d*(*x*,*u*,*t*) is the unknown disturbances of system and is a bounded function.

Because the current *i_α_* and *i_β_* are practically calculated from phase currents *i_abc_*. In the case of only two current sensors (assumed to be ‘a’ and ‘b’ phase), according to literatures [19,20]:

(21) $$\left\{ \begin{array}{l} i_{\alpha }=i_{a}\\ i_{\beta }=\frac{i_{b}-i_{c}}{\sqrt{3}}=\frac{2i_{b}+i_{a}}{\sqrt{3}} \end{array}\right.$$

The effects of the stator αβ axis current sensor fault *f_sα_* and *f_sβ_* are related to the errors in phase ‘a’ and ‘b’ current sensors outputs *f_a_* and *f_b_* as follows:

(22) $$\left\{ \begin{array}{l} f_{s\alpha }=f_{a}\\ f_{s\beta }=\frac{2f_{b}+f_{a}}{\sqrt{3}} \end{array}\right.$$

*e* is the state error defined as $e=\hat{x}-x$, $e_{y_{2}}$ is the output error defined as $e_{y_{2}}={\hat{y}}_{2}-y_{2}$, where, $\hat{x}$ and ${\hat{y}}_{2}$ are the observations of *x* and *y*_2_. The observer based on sliding mode theory is designed as:

(23) $$\left\{ \begin{array}{l} \dot{\hat{x}}(t)=A\hat{x}(t)+Bu(t)+F{\hat{\psi }}_{ext,\alpha \beta }+Dv\\ {\hat{y}}_{2}(t)=C\hat{x} \end{array}\right.$$

where: *v* is the sliding mode correction control signal, and ρ > 0.

(24) $$v=\left\{ \begin{array}{l} -\rho \frac{e}{\left\|e\right\|}\;e\neq 0\\ 0\;e=0 \end{array}\right.$$

The state error equation can be described as:

(25) $$\dot{e}=\dot{\hat{x}}-\dot{x}=Ae+F{\hat{\psi }}_{ext,\alpha \beta }+Dv-F\psi_{ext,\alpha \beta }-Dd=Ae+D(v-d)$$

A non-negative Lyapunov function is given by:

(26) $$V=e^{T}e$$

Then:

(27) $$\begin{array}{l} \dot{V}={\dot{e}}^{T}e+e^{T}\dot{e}={\left[Ae+D(v-d)\right]}^{T}e+e^{T}[Ae+D(v-d)]\\ \;=2e^{T}Ae+2e^{T}D(v-d)=2e^{T}Ae-2\rho e^{T}D\frac{e}{\left\|e\right\|}-2e^{T}Dd\\ \;\leq 2e^{T}Ae-2\rho \left\|e\right\|+2\left\|e\right\|\left\|d\right\|=2e^{T}Ae-2(\rho -\left\|d\right\|)\left\|e\right\| \end{array}$$

Due to the fact *A* is a symmetric negative definite matrix and if we select ρ > 0, then $\dot{V}\leq 0$. Based on the Lyapunov stability theory, *e* converges to zero exponentially. When the unknown input disturbance *d*(*x*,*u*,*t*) is bounded, the sliding mode variable structure observer will generate a special sliding mode motion with the nonlinear discontinuous *v.* Therefore, the system with unknown input is robust on the sliding surface. If the unknown input satisfy the ‘matching condition’, then the invariance of these systems to uncertainties is much more robust than robustness [25].

*r_αβ_*(*t*) is the fault residual defined as:

(28) $$r_{\alpha \beta }(t)=e_{y_{2}}={\hat{y}}_{2}-y_{2}=Ce-Gf_{s}$$

According to Equations (22) and (25), the fault residuals in phase ‘a’ and ‘b’ current sensors can be written as:

(29) $$\left\{ \begin{array}{l} r_{a}=r_{\alpha }=e_{\alpha }-f_{a}\\ r_{b}=\frac{\sqrt{3}r_{\beta }-r_{\alpha }}{2}=\frac{\sqrt{3}e_{\beta }-e_{\alpha }}{2}-f_{b} \end{array}\right.$$

According to Equation (29): when no sensor faults occur, that is, *f_a_* = 0 and *f_b_* = 0, the residual signals *r_a_* = 0 and *r_b_* = 0. When *f_a_* ≠ 0, the residual signal *r_a_* = −*f_a_*. When *f_b_* ≠ 0, the residual signal *r_b_* = −*f_b_*. That is, when sensor faults occur, the residual signal jumps to negative sensor fault value, the residual deviates from the zero values. The fault diagnosis decision rules are described in Table 1, Where: ‘1’ represents *r_i_* ≠ 0, ‘0’ represents *r_i_* = 0.

**Table 1:** Fault diagnosis decision rules.

*r_b_*	*r_a_*	Fault Decisions
0	0	fault free
0	1	fault *f_a_*
1	0	fault *f_b_*
1	1	fault *f_a_*, *f_b_* simultaneously

## 5. Simulations and Analysis

**Figure 1:**
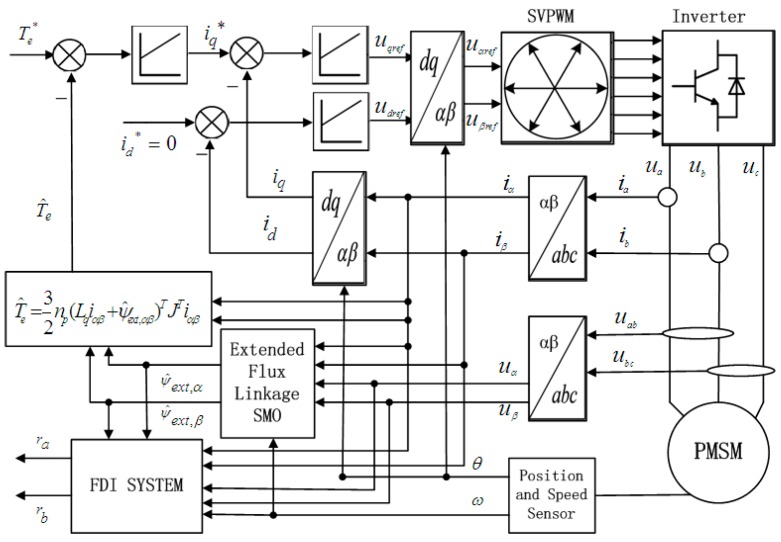
Block diagram of the PMSM driven system under torque closed-loop control with the extended flux linkage SMO and the FDI system.

In this section, simulation results are given based on a sliding mode observer for phase current sensor fault diagnosis. General structure of the simulation setup is shown in Figure 1. In this diagram, the PMSM is controlled by a SVPWM Voltage Source Inverter using a torque closed-loop control strategy and control scheme of $i_{d}^{*}$ = 0.

The parameters of the interior PMSM in this simulation are listed in Table 2. The reference rotor speed and torque are set at 200 rad/s and 500 Nm, respectively. It is assumed that the unknown input disturbance *d* of the system is a random noise, which maximum value is 10 and minimum value is −10. To weaken the chattering of the status variable motion trajectory, the successive approximation function can be used instead of the sign function sgn(·) in the simulation. Three cases are discussed:

(1) No fault condition

**Figure 2:**
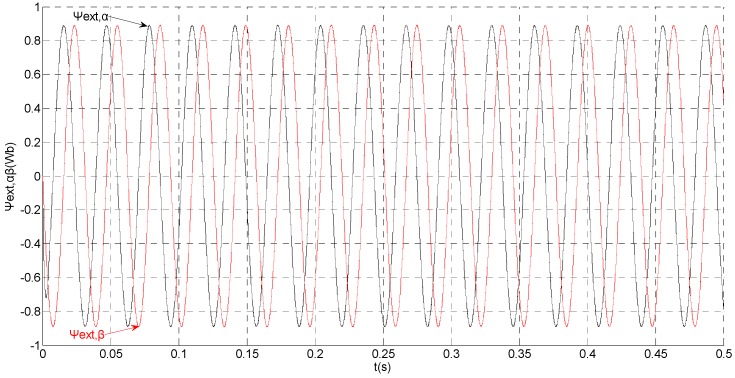
Simulation results of the observation of extended flux linkage.

**Figure 3:**
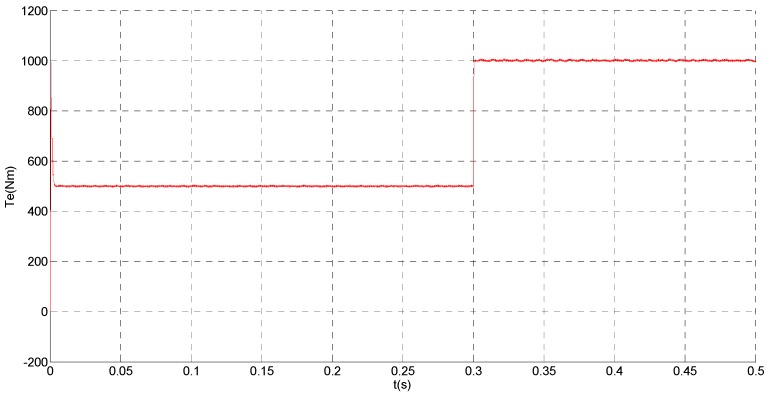
Simulation result of electromagnetic torque.

**Figure 4:**
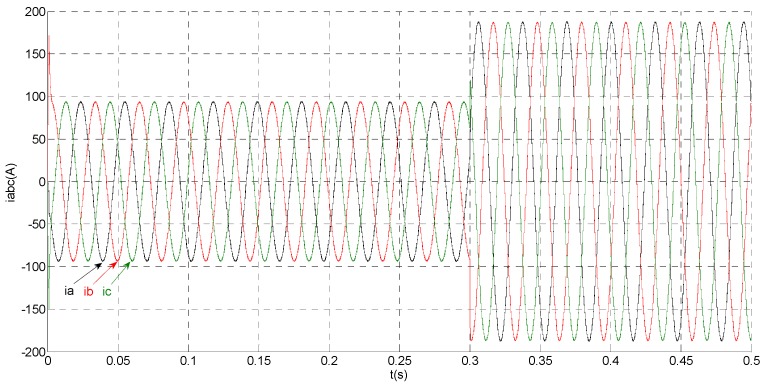
Simulation results of measured phase currents.

**Figure 5:**
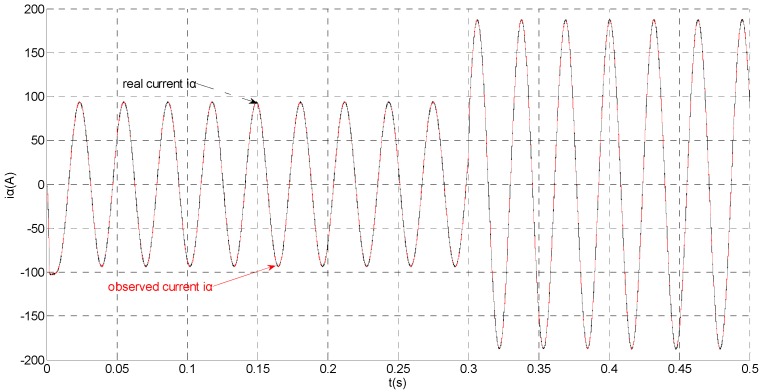
Simulation results of the actual and observation of α axis stator current.

**Figure 6:**
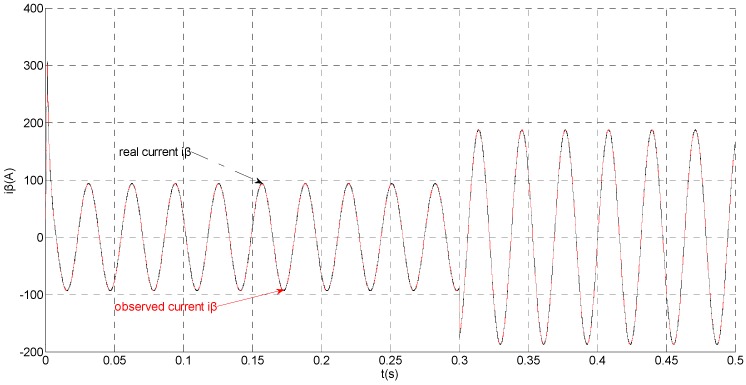
Simulation results of the actual and observation of β axis stator current.

**Figure 7:**
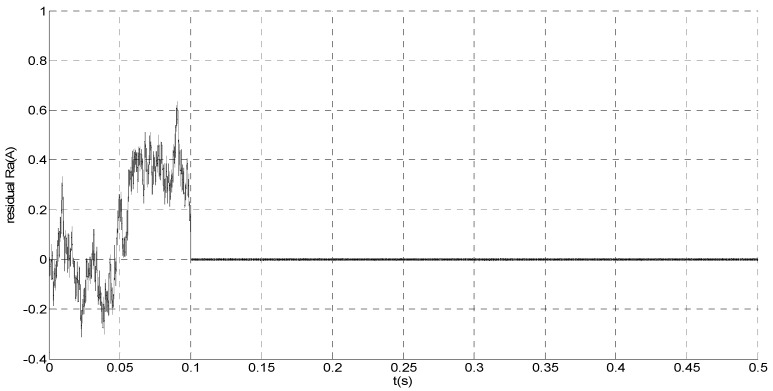
Simulation result of current residual in phase ‘a’.

**Figure 8:**
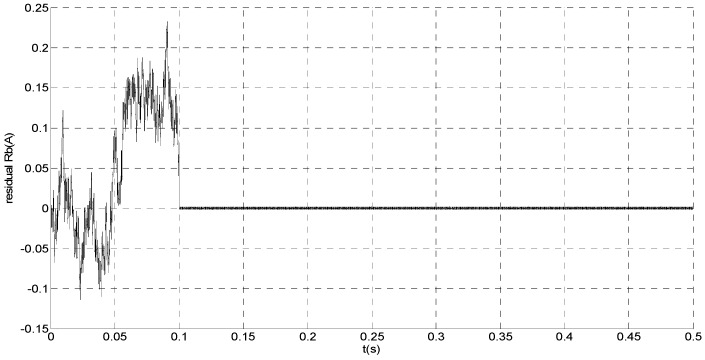
Simulation result of current residual in phase ‘b’.

To evaluate the robustness of the designed SMO, the stator resistance is changed from 0.02 Ω to 0.04 Ω at *t* = 0.2 s, and the torque is changed from 500 Nm to 1000 Nm at 0.3 s. The observation of extended flux linkage is shown in Figure 2. It is insensitive to the variation of stator resistance and torque. The torque calculation value which is obtained by the extend flux linkage is shown in Figure 3. The phase currents are shown in Figure 4, the actual value and the observation of the stator α and β axis currents are shown in Figure 5 and Figure 6, the amplitude of the current is correspondingly larger due to the increase of the torque at *t* = 0.2 s, the phase current and the stator αβ axis currents are insensitive to the stator resistance variation, and the system has good robustness, the observation of stator αβ axis currents can rapidly track the actual value with high precision. The current residuals in phase ‘a’ and in phase ‘b’ are respectively shown in Figure 7 and Figure 8. Furthermore, in order to illustrate the function of sliding mode observer, we introduce signal *v* at *t* = 0.1 s.

**Table 2:** Parameters of interior PMSM.

Parameters	Unit	Value
Stator resistance (*R_s_*)	Ω	0.02
Q axis inductance (*L_q_*)	H	0.003572
D axis inductance (*L_d_*)	H	0.003572
Inertia (*J*)	kg·m^2^	100
Magnetic flux (*ψ_r_*)	Wb	0.892
Number of pole pairs (*P*)	pairs	4
Damping coefficient (*B*)	Nm·s/rad	0.001
DC-bus voltage (*V_dc_*)	V	1500

From Figure 7 and Figure 8, residual is larger when *t* < 0.1 s since the random noise is introduced as the unknown input signal. Then, we must select a larger failure-detection threshold, which may eliminate the detection sensitivity for glitches. However, if *t* > 0.1 s, the disturbance cannot influence the residual since *v* is introduced. Thus, the residual approximates zero, and it is easy to detect the small amplitude faults.

(2) Abrupt gain fault on phase b current sensor

The sensor fault equation can be described as:

(30) $$f_{b}=\left\{ \begin{array}{l} 0\;t\text{<}0.1s\\ -0.5i_{b}\;t\geq 0.1s \end{array}\right.$$

**Figure 9:**
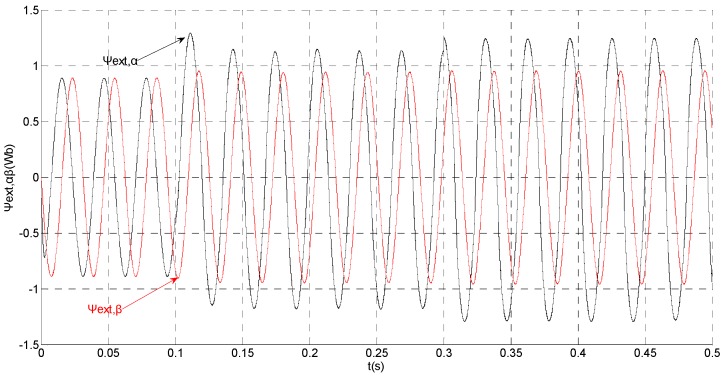
Simulation results of the observation of extended flux linkage.

**Figure 10:**
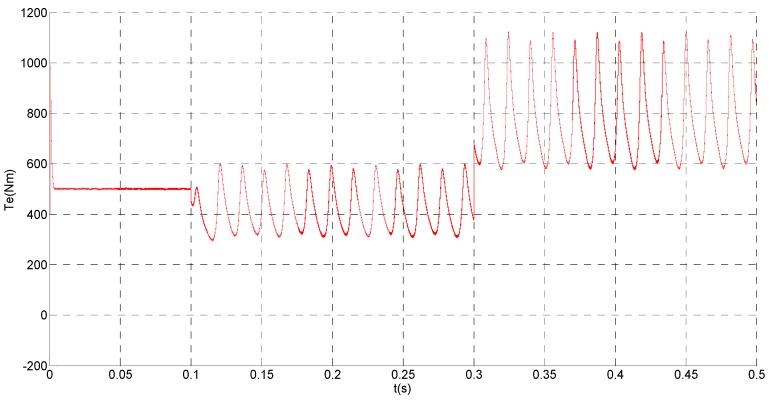
Simulation result of electromagnetic torque.

**Figure 11:**
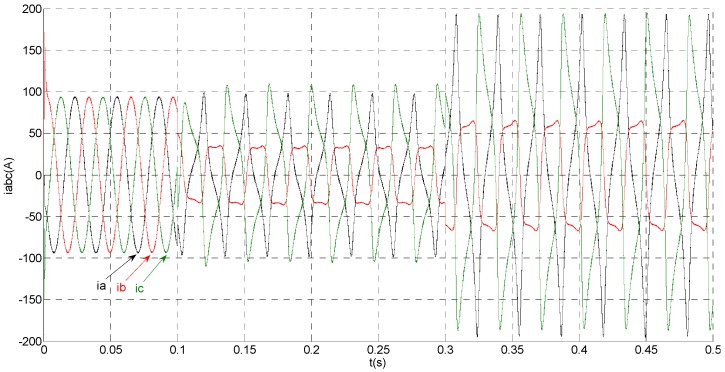
Simulation results of measured phase currents.

**Figure 12:**
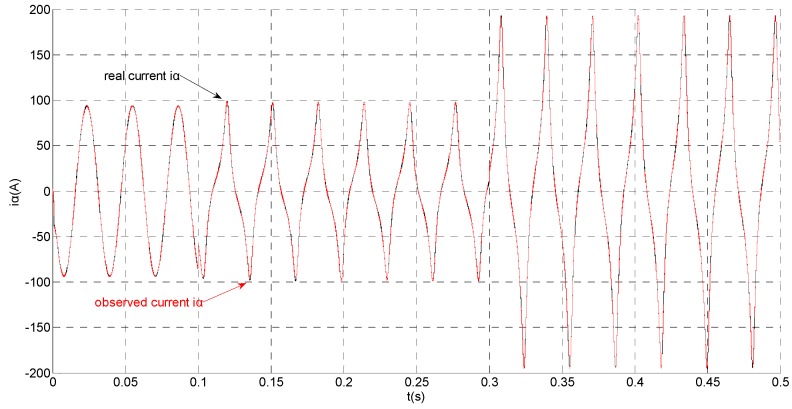
Simulation results of the actual and observation of α axis stator current.

**Figure 13:**
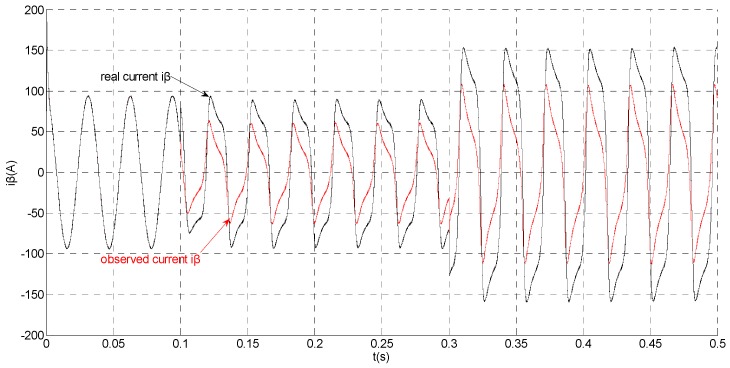
Simulation results of the actual and observation of β axis stator current.

**Figure 14:**
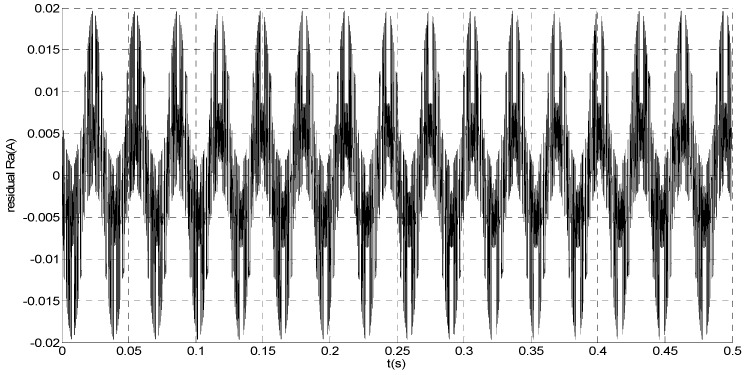
Simulation result of current residual in phase ‘a’.

**Figure 15:**
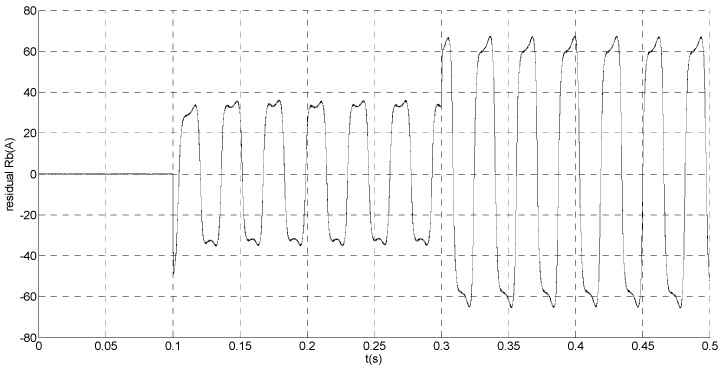
Simulation result of current residual in phase ‘b’.

That is, a negative 0.5 times abrupt gain error is applied to the phase ‘b’ current sensor at *t* = 0.1 s. The torque is changed from 500 Nm to 1000 Nm at 0.3 s. The observation of extended flux linkage and the torque calculation value are respectively shown in Figure 9 and Figure 10. The phase currents, the actual value and the observation of the stator α and β axis currents are shown in Figure 11, Figure 12 and Figure 13, respectively. The current residuals in phase ‘a’ and in phase ‘b’ are respectively shown in Figure 14 and Figure 15. As shown in the following figures, when an abrupt gain fault is imposed, the amplitude of the extended flux linkage increases slightly, the abrupt gain sensor fault produces electromagnetic torque equiamplitude oscillation, the originally balanced current becomes unbalanced, the amplitude of the current in phase ‘a’ and phase ‘c’ increases, the measured current in the b-phase current sensor decreases.

When the torque increases, the amplitude of the current increases at 0.3 s. To the actual value, the observation of the stator β axis current decreases, the residual *r_a_* is closed to zero and the residual *r_b_* is deviated from zero. This suggests that there is no fault on phase ‘a’ current sensor and the faulty phase ‘b’ current sensor is identified.

(3) Abrupt offset fault on phase b current sensor and slow-variation offset fault on phase a current sensor

The sensor fault equations can be described as:

(31) $$f_{a}=\left\{ \begin{array}{l} 0\;t\text{<}0.2s\\ -1.5e^{7t}\;t\geq 0.2s \end{array}\right.$$

(32) $$f_{b}=\left\{ \begin{array}{l} 0\;t\text{<}0.1s\\ -30\;t\geq 0.1s \end{array}\right.$$

**Figure 16:**
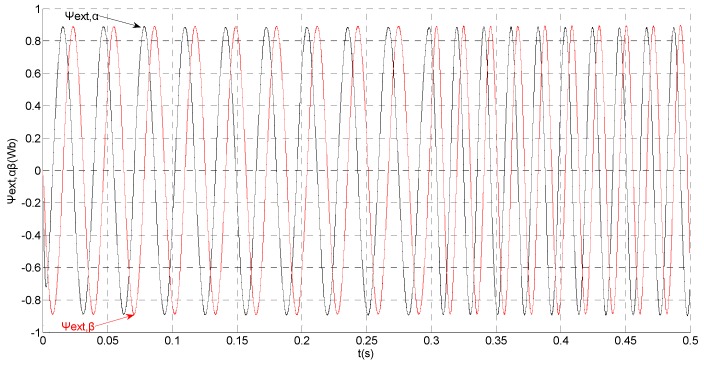
Simulation results of the observation of extended flux linkage.

**Figure 17:**
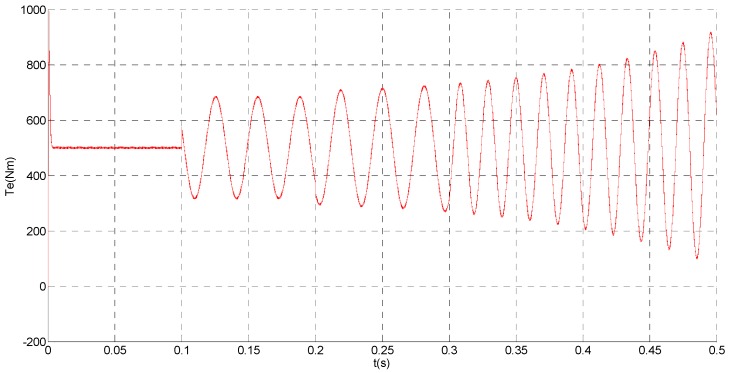
Simulation result of electromagnetic torque.

**Figure 18:**
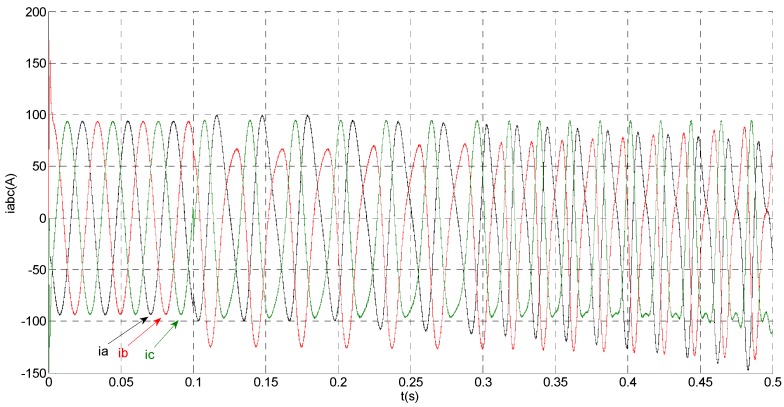
Simulation results of measured phase currents.

**Figure 19:**
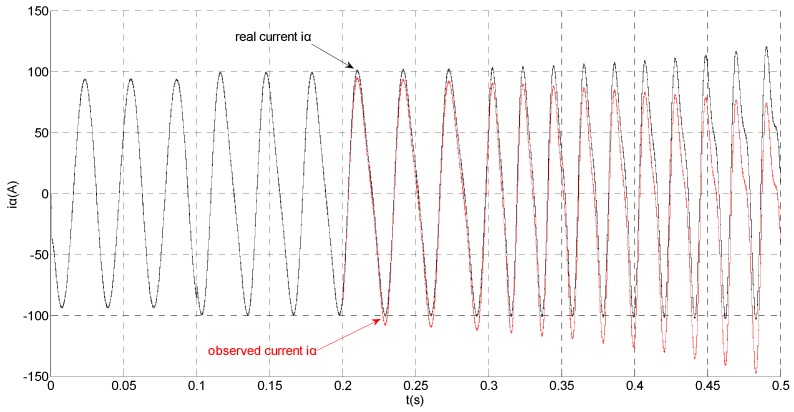
Simulation results of the actual and observation of α axis stator current.

**Figure 20:**
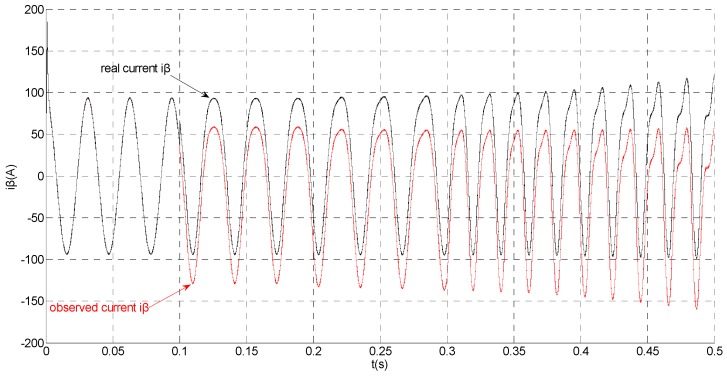
Simulation results of the actual and observation of β axis stator current.

**Figure 21:**
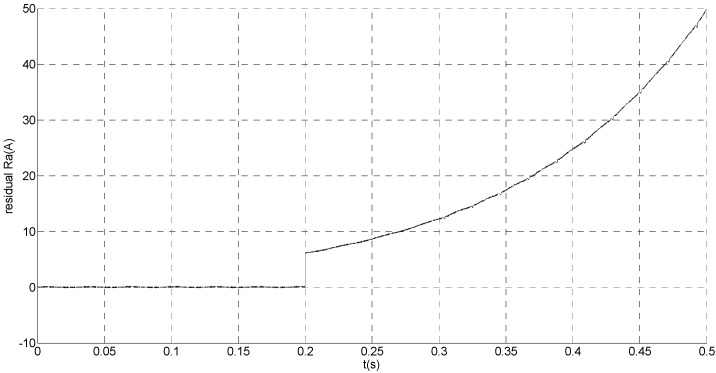
Simulation result of current residual in phase ‘a’.

**Figure 22:**
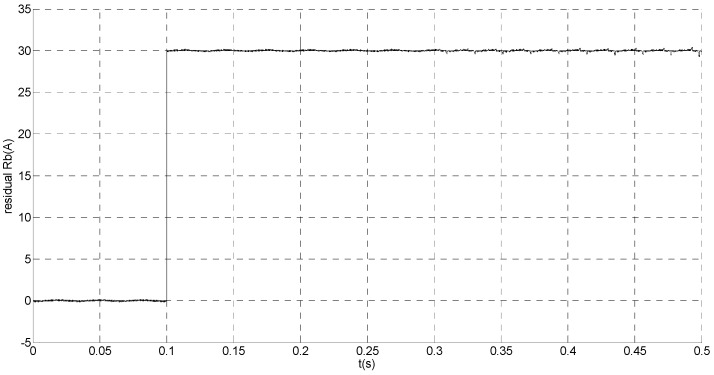
Simulation result of current residual in phase ‘b’.

That is, the current sensor faults are built by adding abrupt offset current of amplitude *f_b_* = −30 to the phase ‘b’ current sensor at *t* = 0.1 s and slow-variation offset current of amplitude *f_a_* = −1.5*e*^7*t*^ to the phase ‘a’ current sensor at *t* = 0.2 s. The value of the speed is changed from 200 rad/s to 300 rad/s at 0.3 s. The observation of extended flux linkage and the torque calculation value are shown in Figure 16 and Figure 17, respectively. The phase currents, the actual value and the observation of the stator α and β axis currents are shown in Figure 18, Figure 19 and Figure 20, respectively. The current residuals in phase ‘a’ and in phase ‘b’ are respectively shown in Figure 21 and Figure 22. As shown in the following figures, when the abrupt offset fault is imposed at *t* = 0.1 s, the amplitude of the extended flux linkage is almost the same since the offset value is relatively small, but it increases slowly after the slow-variation offset fault is imposed at *t* = 0.2 s. The constant offset fault produces an electromagnetic torque equiamplitude oscillation at *t* = 0.1‒0.2 s, but the amplitude of oscillation increases gradually after *t* = 0.2 s. The measured current in b-phase current sensor and the observation of β axis stator current is produced constant offset at *t* = 0.1‒0.2 s, and after *t* = 0.2 s the offset increases gradually. The measured current in a-phase current sensor and the observation of α axis stator current produce a slow-variation offset at *t* = 0.2 s. When the speed increases, the frequency of the current increases at 0.3 s. The residual *r_a_* mutates from zero to 6.0828 at *t* = 0.2 s and then gradually increases exponentially. The residual *r_b_* mutates to 30 at *t* = 0.1 s and then remains constant. This illustrates that the phase ‘a’ current sensor occurs slow-variation fault at *t* = 0.2 s and the phase ‘b’ current sensor occurs abrupt fault at *t* = 0.1 s.

## 6. Hardware-In-The-Loop Experiment and Its Results

**Figure 23:**
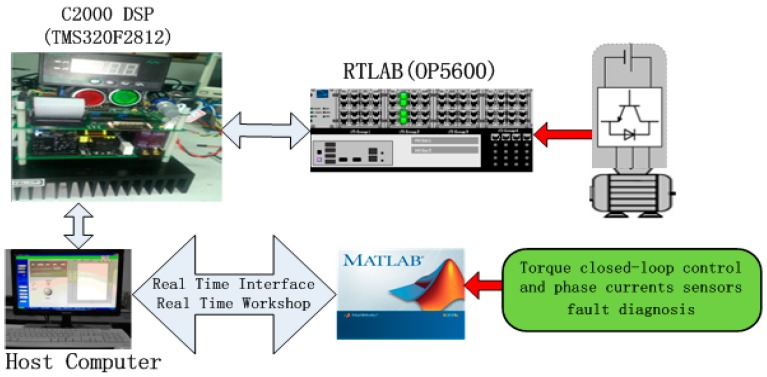
The Structure of RT-LAB hardware-in-the-loop system.

**Figure 24:**
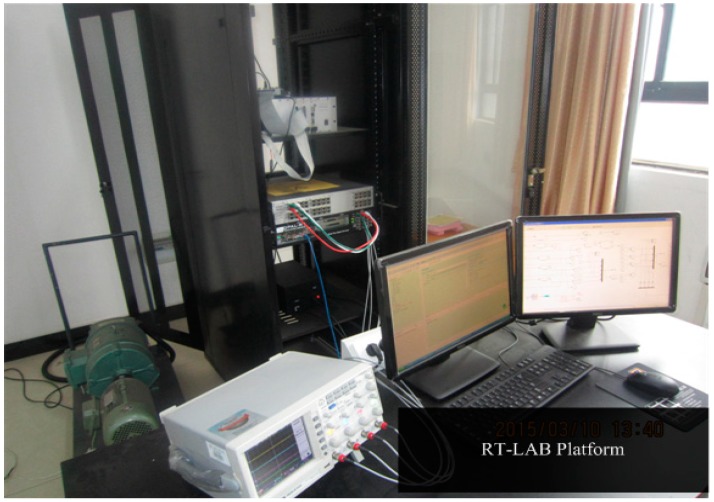
The RT-LAB platform.

**Figure 25:**
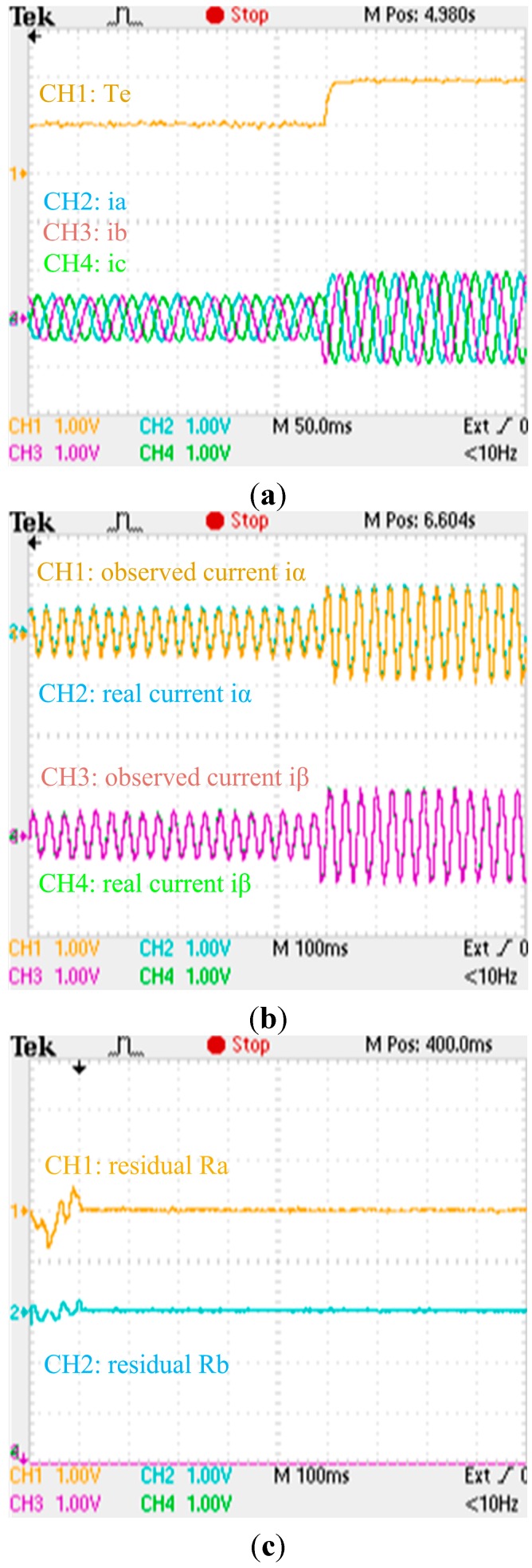
Experimental results when no fault is applied to the phase current sensors: (**a**) Zoom of experimental electromagnetic torque and phase currents responses; (**b**) Zoom of experimental actual and observing stator currents responses; (**c**) Zoom of experimental residual responses. (a) (torque: 500 Nm/div; current: 200 A/div; t: 50 ms/div); (b) (current: 200 A/div; t: 100 ms/div); (c) (residual: 1 A/div; t: 100 ms/div).

**Figure 26:**
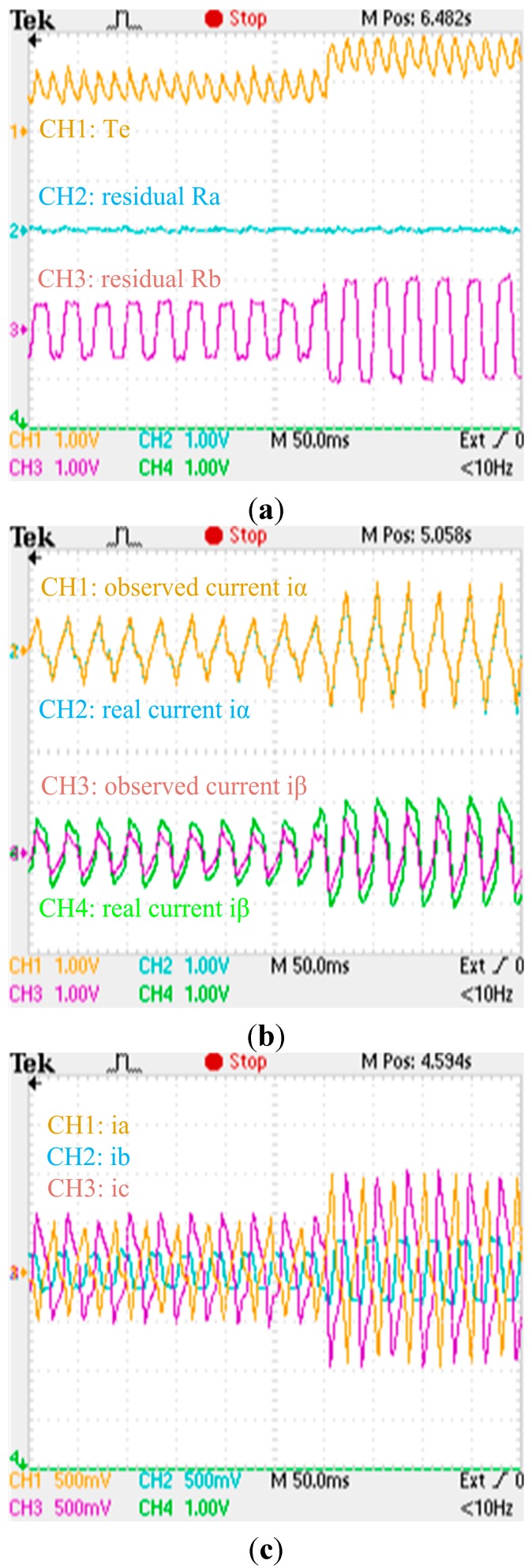
Experimental results when a gain fault is applied to the phase ‘b’ current sensor: (**a**) Zoom of experimental electromagnetic torque and residual responses; (**b**) Zoom of experimental actual and observing stator currents responses; (**c**) Zoom of experimental phase currents responses. (a) (torque: 500 Nm/div; residual: 60 A/div; t: 50 ms/div); (b) (current: 150 A/div; t: 50 ms/div); (c) (current: 100 A/div; t: 50 ms/div).

**Figure 27:**
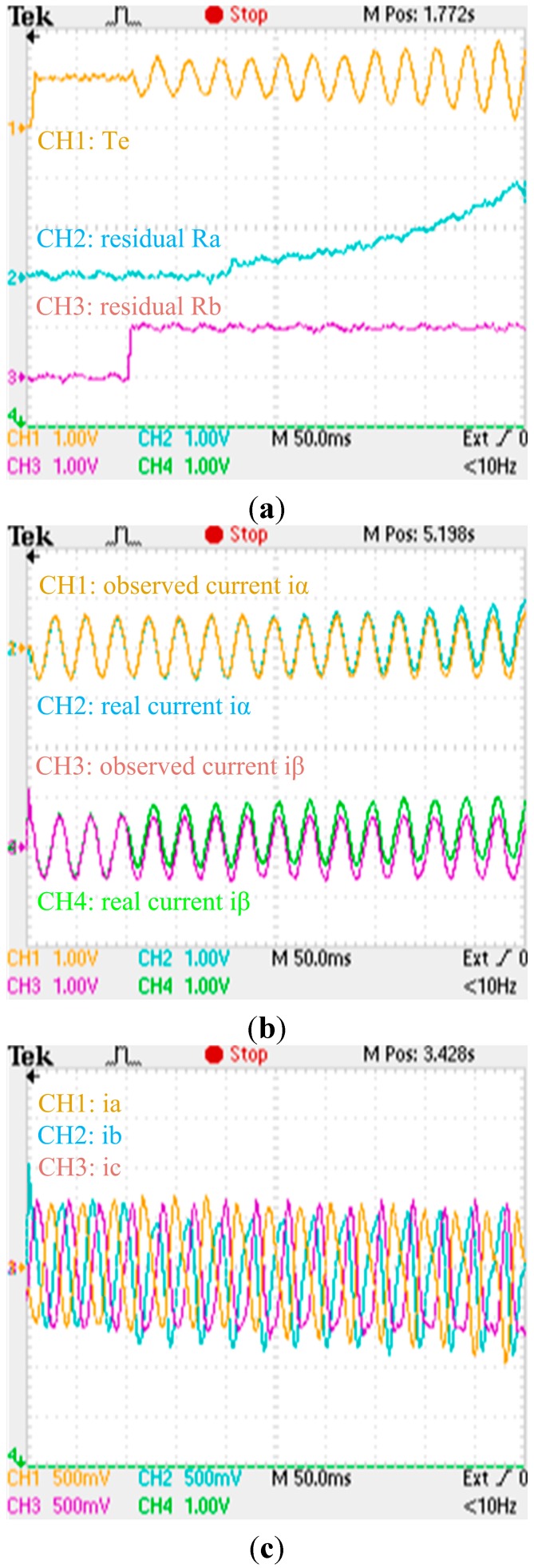
Experimental results when an abrupt offset fault and a slow-variation offset fault are respectively applied to the phase ‘b’ and phase ‘a’ currents sensors: (**a**) Zoom of experimental electromagnetic torque and residual responses; (**b**) Zoom of experimental actual and observing stator currents responses; (**c**) Zoom of experimental phase currents responses. (a) (torque: 500 Nm/div; residual: 30 A/div; t: 50 ms/div); (b) (current: 150 A/div; t: 50 ms/div); (c) (current: 75 A/div; t: 50 ms/div).

To verify the fault diagnosis algorithm based on sliding mode observer, a real-time RT-LAB hardware-in-the-loop system was built and is shown in Figure 23; the RT-LAB platform is shown in Figure 24. It contains a TMS320F2812 DSP controller and RT-LAB OP5600 real-time simulation model (inverter and PMSM). The models of control object (inverter and PMSM) are compiled and then downloaded into the OP5600, and the designed controller model is converted into ‘C’ code and then downloaded into the DSP controller. The analog output signals, such as current signal and position signal of the motor, are collected and then converted to signals with the amplitude of ±10 V, after this, completing the corresponding control algorithm, while six-channel PWM pulses are exported and then sent to the real-time simulation model, the hardware-in-the-loop system of PMSM can be achieved. In this system, the PWM carrier frequency is set at 5 KHz, and the sampling period is set at 20 μs, the parameters of the interior PMSM are listed in Table 2. The experiment results are shown from Figure 25, Figure 26 and Figure 27.

The zooms of experimental electromagnetic torque response, residuals, actual and observing stator currents responses, phase currents responses when no fault is applied to the phase current sensors are shown in Figure 25, respectively. The zooms of experimental electromagnetic torque response, residuals, actual and observing stator currents responses, phase currents responses when an abrupt gain fault is applied to the phase ‘b’ current sensor are shown in Figure 26, respectively. The zooms of experimental electromagnetic torque response, residuals, actual and observing stator currents responses, phase currents responses when an abrupt offset fault and a slow-variation offset fault are applied to the phase ‘b’ and phase ‘a’ currents sensors are shown in Figure 27, respectively. By comparing the simulation results and experimental waveforms under different circumstances, the designed observer can accurately identify the stator currents, effectively identify the faulty sensor for abrupt gain fault and slow-variation offset fault, and is robust to motor parameter changes. The observer structure is concise, and the system has good dynamic performance and high real-time performance.

## 7. Conclusions

An efficient current sensor fault diagnosis algorithm has been presented for the torque closed-loop control system of an interior PMSM using a double observer. First, a sliding mode observer based on the extended flux linkage is designed. It can simplify the motor model, eliminate the salient pole phenomenon and the direct axis inductance dependence, and is used for real-time calculation of the feedback torque. Then a sliding mode current observer is designed in αβ coordinates to generate fault residuals. With the essential feature of sliding mode variable structure technology, being completely insensitive to unknown input disturbances, the generated residuals of the designed fault detection system are not affected by the unknown input, but still maintain high sensitivity to the fault signals. The RT-LAB real-time simulation is used to build a simulation model of hardware in the loop, and the simulation and experimental results show that the method can efficiently identify faulty sensors with abrupt gain faults and slow-variation offset faults. The feasibility and effectiveness of the proposed method are demonstrated. Further research work is being carried out to design a fault tolerant control algorithm to preserve the performance for a faulty PMSM.
